# 

**Published:** 1833-06

**Authors:** 


### MONTHLY LIST OF MEDICAL BOOKS.

[*Medical Works cannot be entered on this List unless a copy be sent for the purpose, the titles of Books having frequently been sent to us as published, which have not appeared for weeks, or even months, after.*]

Part XIX. The Principles and Practice of Obstetric Medicine, in a Series of Systematic Dissertations on Midwifery and on the Diseases of Women and Children. Illustrated by numerous Plates. By D. D. DAVIS, M.D., M.R.S.L., &c.—4to. John Taylor, London.

An Address to the Governors of the Birmingham General Hospital, on the Propriety of appointing Assistant Surgeons to that Institution. By RICHARD MIDDLEMORE, Member of the Royal College of Surgeons, &c.—Birmingham.

A Manual of Pharmacy. By WM. THOMAS BRANDE, F.R.S. &c. Third Edition, corrected and enlarged.—8vo. pp. 544. Renshaw and Rush, London.

The Analysis of Inorganic Bodies. By J. J. BERZELIUS. Translated from the French, by G. O. REES.—12mo. pp. 164. Longman and Co., London.

Observations on the Testicles. By JAMES RUSSELL, F.R.C.S., late Regius Professor of Clinical Surgery in the University of Edinburgh, &c.—8vo. pp. 276. Longman and Co.

No. II. Hortus Medicus; or, Figures and Descriptions of the more important Plants used in Medicine, or possessed of Poisonous Qualities; with their Medical Properties, Chemical Analysis, &c. By GEORGE GRAVES, F.L.S. &c. and JOHN DAVIE MORRIES, M.D. &c. —4to. Longman and Co. London.

This elegant and useful work deserves the support of the profession, and all lovers of botanical knowledge. The engravings are particularly well executed, and the descriptive text is concise and accurate.

An Inquiry into the Causes of Respiration, of the Motion of the Blood, Animal Heat, Absorption, and Muscular Motion; with Practical Inferences. By JAMES CARSON, M.D., Liverpool. Second Edition.—8vo. pp. 447. Longman and Co., London.

Considérations Pratiques sur les Névralgies de la Face. Par — HALLIDAY, Docteur en Médecine des Facultés d'Edinbourg et de Paris.—8vo. pp. 175. A. Pinard, Paris.

### APOTHECARIES' HALL.

**Names of Gentlemen** to each of whom the Court of Examiners have granted Certificates:

MAY 2.

Charles Allsopp Dalby, Ashby de la Zouch
Samuel Smith, Southwold
William Evans, Little Hampton
Maurice Mason, Colchester
Isaac Baruch Toogood, Bridgewater
Wm. Price Jorden, 23, Lower Belgrave street
John Dacie Jeffery, Ottery Sellary, Devon.
Wm. James Jones, Egham, Surrey
Richard Shaw, Tutbury, Stafford
Robt. Palmer Clayton, Oxford st. Manchester

MAY 9.

Edward Grayson, Liverpool

MAY 18.

Nathaniel Barlow, Blackmore, Essex
Wm. Robert Gold Congden, Exeter
James Bailey, Reading
Wm. Grimwood King, London
Wm. Cavendish Lee, Rochester
Matthew Smith Milton, Sunderland
Arthur Squire, London
Edward Wells, Swaffham
James Cropper Weeks, Ramhill, Lancashire

MAY 23.

James Fisher, Wolverhampton
Walter Harsant, Earl Saham
John Hogarth Hedley, Newcastle-on-Tyne
James Haworth Knox, Exeter
John North, Brecon
Henry Peacock
Henry Taylor, Birmingham
George Wm. Wood, Billingshurst.

### METEOROLOGICAL REGISTER,

By Messrs. Harris and Co., Mathematical Instrument Makers, 50, High Holborn.

| | Moon. | Rain gauge. | Thermometer | | | Barometer. | | De Luc's Hygrometer | | Winds. | | Atmospheric Variation. | | |
|---|---|---|---|---|---|---|---|---|---|---|---|---|---|---|
| Apr. | | | 9 a.m. | max. | min. | 9 a.m. | 10 p.m. | 9 a.m. | 10 p.m. | 9 a.m. | 10 p.m. | 9 a.m. | 2 p.m. | 10 p.m. |
| 20 | ● | | 48 | 55 | 46 | 29.88 | 29.90 | 73 | 68 | W | NNW | rain | fine | fine |
| 21 | | | 52 | 56 | 44 | .94 | .94 | 64 | 69 | E | SW | fine | | |
| 22 | | | 48 | 59 | 38 | .99 | .98 | 69 | 69 | W | N | foggy | | |
| 23 | | | 42 | 47 | 48 | .98 | .94 | 69 | 70 | N | W | | | cloudy |
| 24 | | | 55 | 59 | 43 | .78 | .85 | 72 | 72 | ESE | ENE | cloudy | | cloudy |
| 25 | | .10 | 51 | 57 | 43 | 30.02 | 30.04 | 73 | 73 | NE | NE | cloudy | cloudy | fine |
| 26 | | | 49 | 60 | 52 | .02 | 29.94 | 73 | 72 | NNW | NW | fine | fine | fine |
| 27 | ☽ | | 56 | 62 | 47 | 29.82 | .66 | 72 | 70 | WNW | NW | showery | | |
| 28 | | | 51 | 58 | 43 | .58 | .47 | 69 | 68 | WNW | W | cloudy | | |
| 29 | | | 52 | 55 | 43 | .40 | .39 | 68 | 68 | WSW | W | cloudy | | |
| 30 | | | 53 | 59 | 45 | .42 | .48 | 68 | 67 | WNW | NW | | | |
| May 1 | | | 55 | 60 | 52 | .46 | .40 | 67 | 72 | WSW | WNW | fine | | cloudy |
| 2 | | | 55 | 59 | 54 | .30 | .48 | 75 | 77 | SW | SSW | showery | rain | fine |
| 3 | | | 54 | 61 | 57 | .60 | .65 | 81 | 79 | SW | WSW | overcast | fine | |
| 4 | ○ | | 62 | 68 | 58 | .70 | .80 | 75 | 76 | WSW | NNW | fine | | |
| 5 | | | 65 | 70 | 52 | .97 | 30.19 | 65 | 63 | NE | SE | | | |
| 6 | | | 63 | 66 | 50 | 30.24 | .24 | 63 | 61 | ESW | SSE | | | |
| 7 | | | 61 | 65 | 52 | .16 | .01 | 60 | 62 | SE | E | | | cloudy |
| 8 | | | 61 | 67 | 53 | 29.86 | 29.75 | 65 | 65 | ESE | SE | cloudy | | fine |
| 9 | | | 64 | 73 | 53 | .60 | .57 | 65 | 65 | S | WSW | fine | | |
| 10 | | | 62 | 65 | 54 | .75 | .80 | 65 | 65 | WSW | WSW | cloudy | | |
| 11 | ☾ | | 61 | 67 | 57 | .87 | .90 | 65 | 64 | WSW | SE | cloudy | | |
| 12 | | | 65 | 73 | 55 | .85 | .79 | 64 | 64 | SW | SW | fine | | |
| 13 | | | 62 | 71 | 55 | .83 | .89 | 64 | 64 | NW | WNW | | | |
| 14 | | | 64 | 70 | 59 | .97 | .88 | 64 | 63 | W | SSW | | | cloudy |
| 15 | | | 75 | 80 | 65 | .77 | .70 | 62 | 58 | S | S | | | fine |
| 16 | | | 75 | 78 | 62 | .70 | .72 | 55 | 55 | SW | SW | | | |
| 17 | | | 76 | 79 | 61 | .64 | .81 | 56 | 56 | SE | NNW | | | |
| 18 | | | 62 | 68 | 54 | 30.02 | 30.07 | 56 | 55 | NE | SE | cloudy | | cloudy |
| 19 | ● | | 58 | 62 | 54 | 29.94 | 29.96 | 59 | 75 | NE | E | fine | rain | |

The quantity of Rain fallen in April, was 1 inch and 22.100ths.

### INDEX

TO THE

### FOURTEENTH VOLUME OF THE NEW SERIES

OF

### THE LONDON MEDICAL AND PHYSICAL JOURNAL.

---

PAGE

AIR, accidents from entrance of, into veins, during surgical operations . 344
Albinos, queries respecting . 104
Alcohol, proportions of, in different liquors . . . 165
Anatomy, notice of Dr. Grant's lectures on comparative . . 171
Aneurism by anastomosis of the temporal artery . . . 333
of the thyroid gland cured by tying the thyroid arteries . 155
Anemia, pathology of . . 408
Apothecaries' Hall; names of gentlemen passed . 88, 176, 263, 352, 440, 528
regulations respecting dispensaries 261
Company; cause tried, and verdict obtained by, against an Edinburgh physician, for practising pharmacy in England without the licence 349
Army Medical Board, new regulation of, 172
Arsenic, poisoning by . . 83
Ascites, cases of, cured by compression 102
Asphyxia, case of epileptic . 107
Atomic theory . . . . 165
, ancient and modern opinions of the . . 319

Belladonna given to prevent scarlet fever . . . . 518
Bladder, cases of fungus of . 68
Blister, lunar caustic . . 518
Blood, composition of . 257
, essay on diseases, &c. of . 407
Botanical prizes awarded . 84
Botany, Outlines of, notice of Mr. Burnett's . . . 486
Brookes, Joshua, Esq., death of . 172
Bulimia, case of congenital . 418
Burnett, Professor; inaugural address delivered at the Medico-Botanical Society . . . 89, 287

Calculus in urethra of a child, causing retention of urine . . 158
Camphor, case in which a large dose was taken . . . 433
Cancerous disease successfully eradicated by repeated operations . 252

PAGE

Carbonic acid gas, alteration of sound in vessels containing . . 524
Carbonic oxide gas, on preparation of 525
Carotid artery tied . . 512
Cataract, on congenital . . 441
Chlorate of lime successfully used in cases of itch . . . 422
Cholera, Treatise on . 265, 353, 456
, on . . . 25
Colchicum in gout, &c. . . 219
Compression, ascites cured by . 102
Convulsions in children . 339

Defecation, new views of the process of 387
Deformity, singular cases of hereditary 164
Dictionary of Practical Medicine, notice of Dr. Copland's . . 150
Diseases transmitted by electricity . 253

El Malambo, on the febrifuge bark of 450
Electricity, curious experiments on medical . . . . . 253
Embryology . . . 399
Emphysematous tumors of neck, &c. 515
Endowment, differences of natural . 299
Enemas, danger of opiate . 236
Epilepsy, case of, of twenty years' standing, cured . . . 63
Epileptic asphyxia . . 107
Examinations at Edinburgh to be conducted in English instead of Latin 261
Extremities, swelling of, after delivery 283

Factory labour . . 109, 501
Fistula, use of actual cautery in vesico-vaginal . . . . 245
Fœtus in utero, nourished through the medium of lymphatic vessels which pass from the mother . . 368
Fossil flora, on the . . 167
Fracture of femur and rib from disease 39

Gout, on treatment of . . 218
Gregory, Dr. James C., death of . 172
Gunshot wounds, cases of . 417

Heads from New Zealand, their importation prohibited . . 438
Heart, case of hydatids of the . 143

Hemorrhage, new mode of stopping . 162
, fatal, from a single leech-bite . . . . 234
Hemorrhagic disease terminating in purpurea . . . 205
Hereditary deformities . . 164
Hernia, cases of strangulated femoral 197
Hooping-cough, prussic acid in . 243
Homœopathic doctrines, curious letter in favour of . . . 349
Hybernation, on . . . 54
Hydatids of heart, case of . 143
Hydrophobia, new cure for . 86
Hysterical paroxysm, remedy during 245

Iliac artery successfully tied . 434
Infanticide, remarks on . . 1, 370
, value of the hydrostatic test, in cases of . . 1
Inflammation, review of a work on 45
Influenza, account of the, of April 1833 437
Infusoria, marvellous multiplication of 167
Imposture; curious case of a man who had passed for a woman . 168
Intermittent fevers, on the pathology, &c. of . . . . 177
, on bleeding in the cold stage of . . . 189
Intestines, frequency of ulceration of, in phthisis . . . 161
Itch cured by chlorate of lime . 422

Jaundice, composition of the blood in 257
Joberns, Mr., death of . . 86
Juribali, on the . . . 19

King's College, prizes distributed at 526

Labour at factories . . 109, 501
Labour produced by rupturing the membranes; results of the practice in many cases . . . . 82
Leeches, preservation and reproduction of . . . . 256
Leech-bite, death from a single . 234
Leucorrhœa, remedy for . 245
Lithotripsy, remarks on . . 72
Liver, on diseases of . . 209
, insidious advance of diseases of the . . . 214

Malaria, on . . . 15
Malt poultices, utility of . 245
Membranes, result of rupture of the, in many cases, for the purpose of bringing on labour . . . 82
Mental depression, causing phthisis . 70
Mercury, small dose of, causing ptyalism . . . 157
Midwifery, Dr. Waller's half-yearly report of . . . 101
Military Surgery, review of Sir George Ballinghall on . . 494
Milk, portable . . . 166
Morbid Anatomy, notice of Dr. Hope's illustrations of . . 151
Mortality, average, of Europeans . 166
Moxas successfully used in paraplegia 153

Narcotism, fatal, from twelve drops of laudanum injected into the rectum 236
Nerves, notice of Mr. Swan's splendid Demonstrations of . . 228
Nervous system, on the pathology of 423

Opium a cure for ptyalism . 71
Ophthalmic Hospital, report of . 260

Paralysis, Lecture on . . 336
, on strychnine in . 162
Paraplegia without obvious cause, cured by moxas . . . 153
Parturition, remarks on . . 77
in brutes less safe than supposed . . . ib.
not so easy nor expeditious among barbarians as generally imagined . . . . 79
Penis and testes, extirpation of . 252
Phlebitis, uterine . . 311
Phrenologists not fatalists . . 305
Phrenology, contributions to pathological . . . . 395
Phthisis, intestines frequently ulcerated in . . . . 161
, curious account of, caused by mental depression . . 70
Placenta, separation of, followed by fatal hemorrhage . . 331
and uterus, connexion between 365
Plants, secretion of water by certain . 259
Poisoning with arsenic . . 83
Polypus of the uterus . . 384
Prostate gland, remark as to the origin of the third lobe of . . 122
Ptyalism cured by opium . . 71
from a small dose of mercury 157
Prussic acid in hooping-cough . 243
Puerperal fever, on . . 307
Purpura after hemorrhagic disease from venesection . . . 205
Puy de Dôme, account of the ascent of the . . . . 519

Quinine, effects of large doses of . 233

Rectum, on the usual presence of feces in the . . . . 386
Rickets, remarks on . . 137
Rudolphi, M., death of . . 86
Rumford medals . . . 170

Skin, on the structure and functions of the . . . . 145
Smell, loss of, from injury to the first pair of nerves . . . 69
Spasm relieved by ligature . 342
Spine, lateral curvature of the, not arising from rickets . . 141
, case of fractured . 41
Spurzheim, Dr., death of . 173
Stramonium in tic douleureux . 244
Stricture of urethra, analysis of a treatise on . . . 121

Strychnine in paralysis . . 162
Swan, Mr., notice of his splendid "Demonstrations of the Nerves" . 228
Swelling of extremities, after delivery 283

Testes and penis, extirpation of . 252
Tetanus cured by dividing the tibial nerve . . . . 240
Thyroid gland, aneurism of, cured by tying the thyroid arteries . 155
Tibia, compound fracture of the; secondary hemorrhage . . 237
—, case of chronic abscess of . 133
Tic douleureux, stramonium in . 244
Tongue, hypertrophy of the . 250
Tubercle, on the seat of . . 226
—, inoculation from matter of 161
Tumors, observations on, with cases . 35

Umbilical vesicle . . 423
Urethra, analysis of a treatise on . 121
Urethra, calculus in the, causing retention of urine . . 158
Urine, retention of, from calculus in the urethra of a child . 158
Uterus, inflammation and softening of the . . . . . 310
— of the veins of 311
— and placenta, connexion between the . . . 365

Vaccination influenced by temperature 330
Veins, danger from wounds in large . 518
Venereal disease, review of Mr. Wallace on . . . . . 473
Venesection followed by hemorrhagic disease . . . 205
Vesico-vaginal fistula, use of actual cautery in . . . 245

Water secreted by certain plants . 259
Wry-neck remedied by operation . 514

---

COLLECTANEA . . 70, 161, 240, 336, 423, 515

INTELLIGENCE . . 84, 168, 260, 348, 437, 526

METEOROLOGICAL REGISTER 88, 176, 264, 352, 440, 528

### CRITICAL ANALYSES.

Medico-Chirurgical Transactions. Vol. XVII. . . . . . 34
Observations on Tumors, with Cases, by W. Lawrence, Esq. F.R.S. . 35
Some Account of a Case in which the Left Femur and Fifth Rib on the Right Side were fractured, in consequence of Disease. By S. Cooper, Esq. . . . . . 38
Case of Fractured Spine, by Mr. Barlow 41
A Treatise on Inflammations; explaining their Pathology, Causes, Consequences, and Treatment, &c. By G. Rogerson, Esq. . . . 44
On Hybernation, by Marshall Hall, M.D. F.R.S. L. & E. . . . 54
A Treatise on the Urethra, its Diseases, especially Stricture, and their Cure. By Benjamin Phillips, Esq. . 121
Cases of Chronic Abscess of the Tibia, by B. C. Brodie, Esq. . 132
On a Peculiarity in the Conformation of the Skeleton in Rickets, by Mr. Shaw . . . . . 137
Hydatids of the Heart, by Mr. Evans 143
An Essay on the Structure and Functions of the Skin, by Wm. Wood, M.D. 145
A Treatise on Diseases of the Liver, and on Bilious Complaints; with Observations on the Management of the Health of those who have returned from Tropical Climates, and on the Diseases of Infancy. By G. H. Bell, Esq. . . . . . 209
A Further Examination of the Principles of the Treatment of Gout, by Sir C. Scudamore, M.D. F.R.S. &c. Second Edition . . . . . 218
Illustrations of Elementary Forms of Disease, by Robert Carswell, M.D. 224
Researches on the Pathology and Treatment of some of the most important Diseases of Women, by Robert Lee, M.D. F.R.S. . . - 305
An Introduction to the Atomic Theory; comprising a Sketch of the Opinions entertained by the most distinguished ancient and modern Philosophers with respect to the Constitution of Matter. By C. Daubeny, M.D. F.R.S. . 319
New Views of the Process of Defecation, and their Application to the Pathology and Treatment of Diseases of the Stomach, Bowels, and other Organs; together with an Analytical Correction of Sir C. Bell's Views respecting the Nerves of the Face. By James O'Beirne, M.D. . . . 386
Outlines of Human Physiology, by Herbert Mayo, F.R.S. F.G S., Professor of Anatomy in King's College, London, &c. Third Edition . . 399
A Dictionary of Practical Medicine, &c. Part I. By James Copland, M.D. 406
A Treatise on the Venereal Disease, and its Varieties, by William Wallace, M.R.I.A. &c. . . . 473
Outlines of Botany; being a Practical Guide to the Study of Plants. By G. T. Burnett, Professor of Botany in King's College, London, &c. Nos. I. II. and III. . . . 486
Outlines of the Course of Lectures on Military Surgery, delivered in the University of Edinburgh, by Sir Geo. Ballingall, M.D. F.R.S. E. &c. . 494

### BIBLIOGRAPHICAL NOTICES.

A General System of Gardening and Botany, by George Don, F.L.S. . 61
A Dictionary of Practical Medicine, by James Copland, M.D . . 150
Principles and Illustrations of Morbid Anatomy, adapted to the Elements of M. Andral, and to the Cyclopædia of Practical Medicine. By J. Hope, M.D. 151, 230
A Demonstration of the Nerves of the Human Body. Parts II. and III. By Joseph Swan . . . 228
The Principles and Practice of Obstetric Medicine. Parts XIV. XV. and XVI. By D. D. Davis, M.D. &c. . 229
Transactions of the Society for the Encouragement of Arts, &c. No. I. . 230
British Flowering Plants, by W. Baxter 231
English Botany, by C. E. Sowerby, F.L.S. ib.
Medical Botany, by G. T. Burnett . ib.
A Treatise on Indigestion and its Consequences, called Nervous and Bilious Complaints. By A. P.W. Philip, M.D. 328
Principles of Physiological Medicine, by F. J. V. Broussais, M.D. From the French, by Isaac Hays, M.D., and R. E. Griffith, M.D. . . 329
Explanation of the Causes why Cowpox sometimes fails to prevent Smallpox, &c. By Edward Leese, Esq. . 330
A Plain Statement of Vaccination, &c. 416
Observations on Impediments of Speech, with Remarks on their successful Treatment, by Richard Cull . ib.
A Manual of Pharmacy, by Wm. Thos. Brande, F.R.S. &c. . . 514
The Black Death in the Fourteenth Century, from the German of J. F. C. Hecker, M.D. Translated by B. G. Babington, M.D. . . ib.

**Names** *of* **Authors** *of whose Works, Observations, &c. a more or less detailed Account is given in this Volume.*

Anderson, A. Esq., case of Hemorrhagic Disease, following Venesection after Amputation, and finally terminating in Purpura . . . 205
Atlee, E. P. M.D., on Prussic Acid in Pertussis . . . 243
Babington, B. G. M.D., notice of his translation of Hecker's "Black Death in the Fourteenth Century" . 514
Ballard, E. G. Esq., Queries respecting Albinos . . . 104
Remarks on a Case of Paralysis 255
Ballingall, Sir George, review of his "Outlines of Military Surgery" . 494
Barlow, Mr., case of Fractured Spine 41
Baudeus, Prof., cases of Gunshot Wounds 417
Baxter, Mr. W., notice of "British Flowering Plants" . . 231
Bell, Dr. J., on Intermittent Fevers 177
Bell, G. H. Esq., analysis of his Treatise on the Liver . . 209
Blake, Andrew, M.D. M.R.C.S. &c., on Malaria . . . 15
Boswell, J. C. Esq., Lunar Caustic Blister 518
Brande, W. T. F.R.S., notice of his Manual of Pharmacy . . 514
Brande, W. Esq., Proportions of Alcohol in different Spirituous Liquors 165
Brodie, B. C. Esq. F.R.S., on Chronic Abscess of the Tibia . . 133
Broussais, F. J. V. M.D., notice of translation of his Principles of Physiological Medicine, &c. . . 329
Burnett, Prof., Inaugural Address delivered at the Medico-Botanical Society 89, 287
notice of his "Outlines of Botany" . 486
notice of his "Medical Botany" . 231
Emphysematous Tumor of the Face . 517
Carlisle, Sir A., on a new Remedy for Hydrophobia . . . 86
Carswell, R. M.D., review of his "Illustrations of Elementary Forms of Disease" . . . 224
Chiappa, Prof., on Enemata of Iced Water as a Remedy during the Hysteric Paroxysm . . . 245
Christie, A. T., M.D., on Epidemic Cholera . . . 265, 353, 456
Cooper, Samuel, Esq., case of Fractured Femur and Rib from Disease . 39
Copland, James, M.D., notice of his "Dictionary of Practical Medicine" 150, 407
Coulson, W. Esq., cases of Swelling of the Extremities after Delivery . 283
Cull, Richard, notice of his "Observations on Impediments of Speech" 416
Dance, M., case of Paraplegia cured by Moxas . . . . 153
Daubeny, C. M.D. F.R.S., review of his "Introduction to the Atomic Theory" 319
Davies, D. D. M.D., notice of his "Principles and Practice of Obstetric Medicine" . . . 229
Descuret, Dr., case of Congenital Bulimia 418
Dirchoff, M., on Portable Milk . 166
Don, George, F.L.S., notice of his "System of Gardening and Botany" . 61
Duffin, E. W. Esq., case of Epileptic Asphyxia . . . 107
Earle, H. Esq., case of Retention of Urine, and great Tumefaction of the external Parts, produced by a Calculus impacted in the Urethra of a Child . . . . 158
Ehrenberg, C. G., on the Multiplication of the Infusoria . . 167
Eickhorn, G. M.D., case in which a large Dose of Camphor was taken . 433
Elliotson, Dr., case of Poisoning with Arsenic . . . . . 83
Epps, John, M.D., case of Epilepsy 63
Evans, Mr., on Hydatids of the Heart 143
Fantonelli, Professor, Treatment of Itch by Chlorate of Lime . . 422
Fenoglio, Dr., cases of Ascites cured by Compression . . . 102
Foote, J. Esq. L.A.S. A.M. B.S., case of Placental Separation; fatal Hemorrhage . . . . 331
Gensoul, M., on Danger from Wounds of large Veins . . . 518
Grant, Dr., notice of his "Lectures on Comparative Anatomy" . . 171
Graves, Dr. J., Ptyalism cured by Opium 71
Graves, Prof., on the Pathology of the Nervous System . 423
Guthrie, G. J. Esq., his Practice at the Royal Westminster Ophthalmic Hospital . . . . 260
Hall, Dr Marshall, on Hybernation 54
Hall, Dr. J. C., Extirpation of the Testes and Penis, affected with Cancerous Disease . . . . 252
External Iliac Artery successfully tied 434
Hamilton, Dr. Wm., account of the El Malambo . . . 450
Hancock, Dr. John, on the Euribali 19
Heurteloup, Baron, on Lithotripsy 72
Holm, H. H. Esq., Contributions to Pathological Phrenology . 195
Hope, Dr. J. F.R.S., notice of his "Illustrations of Morbid Anatomy" 151, 230
Jones, John, Esq., on Cholera . 25
Kane, Mr., on the Composition of the Blood in Jaundice . . 257
Kennedy, Dr., on the Actual Cautery in Vesico-Vaginal Fistula . . 245
Kopp, Dr., remedy for Leucorrhœa ib.
Laennec, M., curious account of Phthisis caused by Mental Depression . 70

Laennec, M., case of Inoculation from the Matter of Tubercle . . 161
Frequency of Diarrhœa and Ulceration of the Intestines in Phthisis ib.
Lauth, M. E. A., fils, on the Connexion between the Uterus and Placenta, &c. 365
Lawrence, W. Esq. F.R.S., case of Ptyalism, with extensive Swelling of the Tongue, from a small Dose of Mercury . . . . 157
on Tumors . 35
Lee, Robert, M.D. F.R.S., review of his work on the Diseases of Women . 305
Leese, Edward, Esq., notice of his "Explanation of the Causes why Vaccination has sometimes failed to prevent Smallpox," &c. . . . 330
Macfarlane, J. M.D., case of Secondary Hemorrhage after Compound Fracture of the Right Tibia . . 237
Malyn, John, Esq., on Factory Labour 109, 501
Mayer, M., on the Umbilical Vessel 423
Mayo, Herbert, F.R.S. F.G.S., review of the Third Edition of his "Outlines of Human Physiology" . . 399
Middlemore, Mr., Lecture on Cataract 441
Mitchell, J. K. M.D., case of Spasm and severe Pain relieved by Ligature . 342
Moreau, M., on the Preservation and Reproduction of Leeches . . 256
Murray, Dr., "Outline of the Atomic Theory" . . . 165
Murray, Dr. J., case of Tetanus, following Punctured Wound in the Foot, cured by Division of the Posterior Tibial Nerve . . . 240
Nicod, Dr., case of Fungus in the Bladder 68
North, John, F.L.S., Emphysematous Tumor of the Neck occurring suddenly 517
case of Hereditary Deformity . 165
O'Beirne, Dr., review of his work on Defecation . . . . 386
Ollivier, Dr., Emphysematous Tumor of the Neck, accompanied by remarkable Circumstances . . 515
Palmer, J. F. Esq., case of Lobulated Polypus of the Os Uteri . . 385
Philip, A. P. W. M.D. F.R.S. L. & E., notice of the Seventh Edition of his "Treatise on Indigestion" . 328
Phillips, B. Esq., on the Urethra and Stricture . . . . 121
Pott, Dr., case of Tic Douleureux cured by Stramonium . . 244
Rayer, M., case of fatal Narcotism from twelve Drops of Laudanum injected into the Rectum . . 236
Roberton, John, Esq., on Parturition 77
Rogerson, G. Esq., on Inflammation 44
Root, Dr., on Paralysis . . 336
Scudamore, Sir C., on the Treatment of Gout . . . . 218
Shaw, A. Esq., on Rickets, &c. . 137
Sowerby, C. E. F.L.S. &c., notice of "English Botany" . . 231
Stafford, R. A. Esq., cases of Strangulated Hernia . . . 197
Swan, Joseph, Esq., notice of his "Demonstrations of the Nerves of the Human Body" . . . 228
Symes, Mr., Ligature of the Carotid, on account of Hemorrhage from the Ear and Fauces . . . 512
Wry Neck remedied by Operation . . . 514
Taylor, A. S. Esq., account of the Case of a Man who had long assumed the Character of a Woman . . 168
on Infanticide . 1, 370
account of the Ascent of the Puy de Dôme, &c. . . 519
Alteration of Sound in Vessels containing Carbonic Acid Gas 524
on the Preparation of Carbonic Acid Gas 525
Tourtual, Dr., on Sulphur as a Preventative against Measles . . 244
Treviranus, Professor, on Secretion of Water by Plants . . . 259
Turnbull, Dr., on Strychnine in Paralysis . . . . 162
Voisin, M., case of Loss of Smell from Injury to the first Pair of Nerves 69
cases of Hereditary Deformity 164
Wallace, Wm. M.R.I.A., review of his work on the Venereal Disease . 473
Waller, Dr. C., Half-yearly Report of Midwifery Cases . . 101
Warren, Dr. J. C., cases of Admission of Air into the Veins during Surgical Operations . . . 344
Wells, Dr., case of Hypertrophy of the Tongue . . . 250
Williams, Dr., on the use of Malt Poultices . . . . 245
Wood, Dr. Wm., on the Structure and Functions of the Skin . . 145

J. AND C. ADLARD, PRINTERS, BARTHOLOMEW CLOSE.

